# Exploring factors influencing university students' participation in fecal microbiota donation: a descriptive qualitative study

**DOI:** 10.3389/fpubh.2026.1873106

**Published:** 2026-08-19

**Authors:** Shiyu Wang, Yu Zhao, Na Zhang, Siyu Fan, Zhi Li, Zhihao Yang, Weiguo Wang, Zhenming Xu, Fengping Liu

**Affiliations:** 1 Wuxi School of Medicine, Jiangnan University, Wuxi, China; 2 Department of Urology, Suzhou Hospital, Affiliated Hospital of Medical School, Nanjing University, Suzhou, China; 3 Department of Urology, Huishan District People's Hospital, Affiliated Huishan Hospital of Xinglin College, Nantong University, Nantong, China

**Keywords:** donor recruitment, fecal donors, fecal microbiota transplantation, qualitative study, university students

## Abstract

**Aim:**

To investigate the motivations and factors influencing university students' decisions to donate feces, and to provide recommendations to hospitals conducting fecal microbiota transplantation (FMT) on specific actions to encourage students to participate in fecal donation.

**Design:**

Qualitative descriptive study.

**Methods:**

In-depth interviews were conducted with fecal donor volunteers using a semi-structured interview approach. Data were analyzed using NVivo 11.0 qualitative analysis software, following the methodology of descriptive qualitative research.

**Results:**

This study interviewed 30 college students and identified two themes and 11 sub-themes: motivations for fecal microbiota donation and barriers to participation. The primary incentives for fecal donors participating in the FMT program included financial incentives, a desire to help others, curiosity about FMT and its therapeutic benefits, the appeal of free health examinations, and the opportunity to support scientific research. Obstacles to participation included negative perceptions, disruption of normal life, overly stringent requirements for FMT donors, psychological barriers and discomfort with stool collection, cumbersome transportation procedures, and a lack of timely and comprehensive feedback on medical checkups.

**Conclusion:**

Addressing obstacles such as negative perceptions, disruption to normal life, stringent donor requirements, psychological discomfort, cumbersome transportation, and insufficient medical feedback is crucial for improving fecal donor recruitment and retention for FMT. Enhancing FMT awareness, emphasizing its benefits, and offering appropriate financial incentives may help motivate university students to donate. Implementing these strategies supports effective donor recruitment and management, advancing FMT therapy.

## 1 Introduction

Fecal microbiota transplantation (FMT) is an innovative therapy that involves transferring gut microbiota from healthy human feces into the gastrointestinal tract of patients (1). This process aims to restore a balanced gut microbiota, treating various intestinal and extraintestinal diseases (2, 3). FMT has demonstrated effectiveness in alleviating gastrointestinal conditions such as recurrent Clostridioides difficile infection (CDI) (4), inflammatory bowel disease (2), irritable bowel syndrome (5), and hepatic encephalopathy (6). Moreover, it has shown potential in several extraintestinal conditions such as metabolic syndrome (7), diabetes (8), cancer (9), and Parkinson's disease (10), linking gut dysbiosis to multiple systemic health issues.

Ensuring the safety and efficacy of FMT necessitates thorough screening of donors. This includes extensive examinations of donors' blood and stool samples to detect transmissible diseases, pathogens, parasites, and other health conditions that could affect the gut microbiota (11). The complex and stringent donor selection process, aimed at maintaining high safety standards, often deters potential donors and complicates recruitment efforts.

The success of FMT hinges on the quality of the donor microbiota. While recipient factors such as genetic makeup and immune status influence FMT outcomes (12), these are beyond the control of medical interventions. Consequently, researchers focus on donor-related factors to optimize FMT success. Despite established screening criteria, actual donor qualification rates remain low, ranging from 0.8% to 31% in various studies (13). For example, a major fecal bank in the US pre-screened over 15,000 candidates, with only 3% qualifying as donors (14). Many candidates were excluded due to factors such as social history and high body mass index (15).

In China, university students may represent a potentially accessible population for fecal donation programs because of their relatively young age, concentrated living environments, and suitability for repeated health assessment and follow-up. Nevertheless, recruiting university students should not be understood as a universal or standardized model across China. Rather, it represents a practical approach adopted by some institutions according to local access to student populations and the organizational conditions of individual donor programs.

Young adults in China may also face social and psychological barriers that extend beyond lifestyle eligibility. Stool donation is often associated with embarrassment, discomfort, and concerns about social judgement, as feces are commonly perceived as private and unpleasant. Previous studies have shown that stigma, disgust, and awkwardness surrounding stool donation can reduce willingness to participate among potential FMT donors (16, 17). Decisions may also be influenced by family members, peers, and prevailing attitudes toward bodily-material donation. In addition, limited public awareness of FMT, concerns about privacy and biological information, uncertainty regarding compensation, and inconvenience associated with repeated screening and transportation may discourage participation. Although studies of blood and biospecimen donation have identified altruism, health benefits, and financial compensation as important motivations (18), the motivations and barriers specific to fecal donation among Chinese university students remain insufficiently understood.

Understanding these motivations is crucial for developing effective strategies for donor recruitment and retention. Factors like lifestyle, diet, exercise, and sleep habits significantly influence gut microbiota composition and, subsequently, the quality of donated feces (18–23). Therefore, it is essential to comprehend the motivations and behavioral patterns of potential fecal donors to ensure a consistent and high-quality donor pool.

Successful donor recruitment depends not only on medical eligibility but also on individuals' willingness to participate and their perceptions of stool donation. University students' willingness to donate stool may be influenced by altruism, financial compensation, access to health screening or health information, and the perceived convenience of the donation process. Conversely, repeated screening, time and transportation requirements, privacy concerns, disgust or embarrassment associated with handling stool, concern about social judgement, and limited knowledge of donor eligibility and donation procedures may discourage participation. Understanding these motivations and barriers is therefore essential for developing feasible and culturally appropriate donor recruitment strategies. Through qualitative interviews, this study explores the motivations and factors influencing university students' decisions to donate feces and provides practical recommendations for hospitals and stool banks to improve donor recruitment, communication, and retention, thereby supporting the sustainable implementation of FMT.

## 2 Materials and methods

### 2.1 Research target

Eligible fecal donation volunteers were approached by members of the research team through WeChat and were invited to participate after receiving an explanation of the study purpose and interview procedures. Interviews were conducted between January 2024 and May 2024 using purposive sampling based on the maximum variation principle. To ensure sample diversity and obtain rich and informative data, students from various schools, disciplines, ages, and genders were selected at Jiangnan University. Inclusion criteria included complete engagement in the fecal donation process, and voluntary participation. Exclusion criteria included failure to complete the whole fecal donation process. The sample size was determined by data saturation, with recruitment continuing until no new relevant information, codes, or themes emerged. The study was reviewed and approved by the Medical Ethics Committee of School of Medicine, Jiangnan University (Y-9). All eligible volunteers who were approached agreed to participate, and no participant withdrew after enrolment.

### 2.2 Research methodology

A qualitative descriptive methodology grounded in naturalistic inquiry was used to explore university students' experiences, motivations, and perceived barriers related to fecal microbiota donation. This approach was selected because it enables a comprehensive yet low-inference description of participants' perspectives in language that remains close to the original data. Interview data were analyzed using inductive thematic analysis (24).

### 2.3 Data collection

A semi-structured interview guide consisting primarily of open-ended questions and appropriate probes was developed in accordance with the study objectives. Following each in-depth interview, an analytic memo was promptly prepared to document preliminary impressions, contextual observations, and emerging analytic insights. Data gathering and analysis were coordinated and integrated. An initial interview framework was developed with the study's objectives in mind, and adjustments were made following a pre-interview to finalize the outline. The interviews were conducted by Shiyu Wang, Yu Zhao, and Siyu Fan, all of whom were female postgraduate students at the time of the study. Before data collection, all three interviewers received training in qualitative research methods and semi-structured interviewing from Fengping Liu. Fengping Liu holds a PhD in medicine, is a female clinical faculty member, and has approximately 10 years of experience in clinical and qualitative research. The training was intended to ensure consistency in interview procedures, the appropriate use of open-ended and probing questions, and sensitivity to participants' responses during the interviews.

Interview questions included: demographic information about the volunteers; their perceptions and attitudes toward FMT; motivations for volunteering; perceived benefits and advantages; the role of these benefits in decision-making; concerns or worries about becoming a donor; factors that increased concern levels; sources of advice or information consulted; involvement of healthcare professionals in decision-making; expectations and goals for becoming a donor; and other motivations or concerns about fecal donation (Table 1).

**Table 1:** Interview questions for participants.

Questions
1. How do you perceive fecal microbiota transplantation?
2. Why did you decide to become a fecal donor?
3. What potential benefits or advantages do you see in fecal transplants?
4. Have you encountered any concerns or worries when considering becoming a fecal transplant donor?
5. Did you seek the advice or counsel of others in making the decision to become a fecal transplant donor? How did you feel about it?
6. What other sources of information did you refer to in making your decision?
7. What are your expectations and goals for becoming a fecal transplant donor?
8. What do you think a qualified or good fecal donor should have?

Face-to-face interviews were conducted in familiar settings selected by the participants, including meeting rooms, classrooms, and private dining booths in campus cafeterias, with efforts made to ensure comfort and privacy. Of the 30 interviews, 28 were conducted face-to-face and two were conducted by telephone. The telephone interviews were conducted with participants S4 and S11 (Table 2). Participants interviewed by telephone were asked to choose a quiet and private setting to minimize interruptions and protect confidentiality. All interviews were conducted one-on-one, and the interviewers flexibly adjusted the order and wording of questions according to participants' responses while maintaining the core interview guide. No repeat interviews were conducted. No individuals other than the participant and the interviewer were present during either the face-to-face or telephone interviews. With participants' permission, all face-to-face and telephone interviews were audio-recorded in full using a KDDI SR702 Smart Recorder. The interviews lasted approximately 40–60 min.

**Table 2:** Basic information of the participants.

ID	Genders	Age (years)	Duration of stool donation (months)	Interview mode
S1	Male	20	10	Face-to-face
S2	Female	24	8	Face-to-face
S3	Female	21	8	Face-to-face
S4	Male	20	7	Telephone
S5	Male	20	7	Face-to-face
S6	Female	23	8	Face-to-face
S7	Female	23	10	Face-to-face
S8	Female	21	6	Face-to-face
S9	Female	21	4	Face-to-face
S10	Female	20	4	Face-to-face
S11	Female	18	12	Telephone
S12	Female	22	6	Face-to-face
S13	Female	19	4	Face-to-face
S14	Male	21	3	Face-to-face
S15	Female	23	3	Face-to-face
S16	Female	23	2	Face-to-face
S17	Female	21	2	Face-to-face
S18	Male	24	12	Face-to-face
S19	Female	22	2	Face-to-face
S20	Male	21	2	Face-to-face
S21	Female	23	3	Face-to-face
S22	Female	23	3	Face-to-face
S23	Male	19	2	Face-to-face
S24	Female	24	2	Face-to-face
S25	Female	21	2	Face-to-face
S26	Female	22	2	Face-to-face
S27	Female	20	1	Face-to-face
S28	Female	23	1	Face-to-face
S29	Female	22	1	Face-to-face
S30	Female	22	1	Face-to-face

### 2.4 Data analysis

Data collection and analysis were coordinated throughout this study. Within 24 h of each interview, the interviewer transcribed the audio recording verbatim. The transcripts were imported into NVivo 11.0 for data management and analysis. Four researchers—Shiyu Wang, Yu Zhao, Siyu Fan, and Fengping Liu—participated in the coding and thematic analysis. The three postgraduate researchers independently conducted the initial coding of the transcripts. The preliminary codes, categories, and themes were then reviewed jointly with Fengping Liu, who supervised the qualitative analysis. Coding decisions were compared through repeated examination of the original transcripts. Any discrepancies in code definitions, data interpretation, or theme assignment were discussed among the four researchers until consensus was reached. When consensus was not immediately reached, Fengping Liu facilitated further review of the relevant transcript passages, and a final consensus was reached collectively. No formal inter-rater reliability statistic, such as percentage agreement or Cohen's kappa, was calculated because the analysis emphasized iterative discussion and interpretive consensus.

The data in this study were analyzed using a thematic approach (24). The analysis was primarily inductive. Codes and themes were derived from the interview data rather than identified in advance or imposed through a predetermined coding framework. Existing literature on medical and biospecimen donation was used to support interpretation of the findings but was not used as an a priori coding framework. The analysis involved the following steps: (1) repeated reading of the interview text to familiarize with the data; (2) initial coding of the relevant, recurring words or sentences; (3) searching for themes by grouping similar content or nature; (4) reviewing themes to ensure coherence; (5) defining and naming themes; and (6) generating a report. Subsequently, the interview data were grouped into thematic frameworks through thorough and iterative comparison with the original data.

Throughout the analysis, the research team assessed whether additional interviews generated new information, codes, or themes. Data saturation was considered to have been reached when no new relevant information or themes emerged. After coding was completed, the research team repeatedly reviewed the transcripts and coding framework, refined the themes, and prepared reflective and analytic memos. Early ideas regarding possible patterns and their relationships to the research questions were also documented. During theme development, the team examined less common, divergent, and contrasting perspectives to ensure that the final thematic structure reflected the diversity of participants' experiences rather than only the most frequently reported views.

### 2.5 Ethical considerations

The goal, subject matter, and interview methodology of this study were all disclosed to potential participants during the recruitment process. Participants were informed that the interviewers were postgraduate researchers and members of the study team. They were told that the purpose of the study was to understand university students' motivations, experiences, and perceived barriers related to fecal microbiota donation, and that the findings would be used to inform future donor recruitment and management. The interviewers' personal views, assumptions, or individual research goals were not disclosed to the participants. In particular, the data collection form, confidentiality policies, and respondents' rights to decline participation or withdraw from the interview were all thoroughly explained. All interviews took place with the study participants' informed consent, which they provided by signing an informed consent form. Furthermore, the researchers de-identified all participant information during transcription and maintained the confidentiality of personal data. Lastly, the researcher's encrypted personal computer was the sole place where the interview data and demographic data from this study were maintained, while the paper data were retained in the researcher's possession.

### 2.6 Rigor

Rigor was established according to the criteria proposed by Lincoln and Guba (31) (25), including credibility, dependability, confirmability, and transferability. The research team enhanced the credibility of the study through peer review. Interview transcripts were returned to all participants for member checking. Participants were invited to review the transcripts, verify the accuracy of their statements, and clarify any ambiguous information. The final themes and synthesized findings were not returned to participants for further validation. No participant requested substantive modifications to the transcripts. Multiple researchers participated in the analysis process to enhance the credibility and confirmability of the findings. In addition, the use of team analysis and topic extraction methods has minimized the bias of research results to the greatest extent possible (26). In addition, there is no prior connection between the interviewer and the participants, and a preparatory interview was conducted with the interviewer before the formal interview to clarify their views on the research question. Throughout the entire data analysis process, researchers conducted in-depth reflections on each step.

The research team acknowledged that their academic and professional backgrounds, as well as their interest in fecal microbiota donation and donor management, might have influenced the interview process and interpretation of the data. Because the interviewers were postgraduate students working under the supervision of a clinical medicine faculty member, participants might have perceived the research team as having professional or institutional authority, which could have encouraged socially desirable responses. To minimize this potential influence, the interviewers used neutral and open-ended questions, avoided expressing approval or disapproval of participants' responses, and encouraged participants to describe both positive and negative experiences. Reflexive discussions and analytic memoranda were used throughout data collection and analysis to examine the potential influence of the researchers' assumptions and perspectives.

This study was reported in accordance with the Consolidated Criteria for Reporting Qualitative Research (COREQ) checklist for studies involving interviews and focus groups.

## 3 Findings

### 3.1 Demographic characteristics

A total of 30 university students participated in the study and completed the interviews (Table 2). The sample comprised 23 women and 7 men aged 18–24 years, including both undergraduate and postgraduate students. Analysis of the 30 interview transcripts identified two primary themes and 11 sub-themes: motivations for participating in fecal microbiota donation and barriers to participation (Table 3).

**Table 3:** Themes and sub-themes derived from the qualitative interviews.

Major theme	Sub-theme
Motivations for participating in fecal microbiota donation	Interest in FMT and its potential benefits
	Motivation to help others
	Economic incentive
	Free health checkups are attractive
	Contributing to scientific research
Barriers to participation in fecal microbiota donation	Negative perceptions
	Impact on normal life
	Stringent donor eligibility requirements
	Psychological barriers and discomfort with stool collection
	Cumbersome transportation procedures
	Insufficient feedback on medical examinations

### 3.2 Theme 1: motivations for participating in fecal microbiota donation

#### 3.2.1 Interest in FMT and its potential benefits

Numerous respondents expressed curiosity about the FMT procedure and a desire to further their understanding of FMT through participation in fecal donation.

*S5 (male, 20 years): “I am very interested in FMT as a therapeutic approach. I knew very little about the field prior to this event and was intrigued by the uniqueness of FMT and was eager to learn more about its principles and the positive changes it could bring to patients. It was this curiosity that drove me to participate in this offering, hoping to gain a more comprehensive understanding of this treatment.”*

*S7 (female, 23 years): “I was shocked to discover about fecal transplants in my teacher's lesson since, up until then, we had never given human excrement any thought. I want to enroll in this program because I find it extremely intriguing and believe it has the potential to be highly significant.”*

*S20 (male, 21 years): “I am interested in whether FMT can be applied to humans. Although most of us know that FMT can be tested on animals, we know very little about whether it can be applied to humans. I have heard that (FMT) can be used to treat a variety of diseases, including depression and insomnia… I am a researcher in bioengineering. Therefore, I would like to know if FMT is indeed a viable medical treatment.”*

#### 3.2.2 Motivation to help others

Numerous respondents stated that they wanted to donate stool to help patients, which demonstrates a compassionate and socially responsible perspective.

*S8 (female, 21 years): “I have always had a heart for helping others. Whenever I come across a person or animal in need of assistance in my life, I always feel sympathetic and willing to lend a hand. Therefore, when I found out about the volunteer recruitment, I felt very excited because even though I am not a healer, I still have the opportunity to provide help to the patients, which makes me feel great.”*

*S13 (female, 19 years): “To start with, I believe that volunteers are wonderful and noble people. Secondly, I was glad to take advantage of the chance to volunteer for fecal microbiota transplantation that was presented to me. I'm thrilled to be doing it. I have the ability to assist many individuals in need. I didn't think twice about joining the program because I want to be one of the many people who commit their lives to the advancement of medicine in daily life, such as physicians, nurses, and other volunteers who work tirelessly to rescue patients.”*

#### 3.2.3 Economic incentive

Financial subsidies were mentioned directly by a few interviewees as a major factor in their decision to engage in volunteer work.

*S9 (female, 21 years): “My decision to come and volunteer was largely driven by compensation. As a student, I don't have a steady source of income, so the lure of money is quite strong for me.”*

*S29 (female, 22 years): “This subsidy appealed to me the most when I first came to volunteer since it would allow me to somewhat raise my living expenses while also relieving some of my family's financial burden.”*

#### 3.2.4 Free health checkups are attractive

For other respondents, the program's free health checkup was a great way to learn about and take care of their health.

*S3 (female, 21 years): “The opportunity for a free medical checkup was extremely appealing to me, and considering the current cost of hospital checkups, I thought it was certainly an excellent opportunity.”*

*S10 (female, 20 years): “I was able to learn more about the condition of my intestines during a physical exam, which resulted in a 70-page report that was quite thorough. I was very interested in that.”*

*S16 (female, 23 years): “During the donation period, the hospital also provides sporadic medical check-ups to volunteers, which serves to further aid in our comprehension of any and all alterations in our physical state…”*

#### 3.2.5 Contributing to scientific research

A few of the interviewees stated that they hoped to advance science and medicine by participating in such events and contributing to scientific research.

*S8 (female, 21 years): “As I arrived to take part in this activity, I also considered how I could help advance the medical cause. I hoped that more patients would benefit from the development of intestinal micro-ecology by feeling better and that it would advance science and comprehensively.”*

*S25 (female, 21 years): “I hope to contribute to the development of medicine by taking part in this volunteer program.”*

### 3.3 Theme 2: barriers to participation in fecal microbiota donation

#### 3.3.1 Negative perception

Participants reported that negative opinions or cautionary advice from family members and friends could discourage them from participating in fecal donation. Limited knowledge of and interest in FMT within their social networks further reduced perceived support for participation.

*S11 (female, 18 years): “Before I decided to take the plunge into volunteering, I did feel apprehensive about the potential leakage of personal genetic information - after all, it was a sample of my bodily fluids that was involved, and the possible security risks made me a little shaky inside.”*

*S16 (female, 23 years): “I was a little concerned about the safety of this at the time because there was a movie about information leakage from blood donations outside that was quite popular at the time. People around me also advised me to be cautious about getting involved.”*

#### 3.3.2 Impact on normal life

Before participating in fecal donation, volunteers frequently had reservations, and some respondents thought that the donation process could create scheduling conflicts.

*S3 (female, 21 years): “Time is probably a factor for me; I need to take the time to save this material and send it along… So, I think time is a test for me.”*

*S23 (male, 19 years): “Regular health exams and screenings are required as part of the fecal transplant procedure, which can cause scheduling conflicts.”*

#### 3.3.3 Stringent donor eligibility requirements

Certain respondents believed that the FMT standards were excessively stringent for donors, making it challenging for them to comply.

*S7 (female, 23 years): “I believe that the criteria during the donation period are quite stringent, including exercise and sleep…I find these rigors challenging.”*

*S15 (female, 23 years): “The prerequisites for fecal donation are far more stringent than those for blood donation. You must fulfill several conditions for fecal donation, starting with the stool test and concluding with the blood sample…The previous time, I had seven or eight tubes of blood collected in addition to fecal and urine tests, all of which had to be essentially inside tolerance. It will be challenging for me to fulfill the requirement if I have to take examinations frequently.”*

#### 3.3.4 Psychological barriers and discomfort with stool collection

Some of the respondents said that the entire procedure of collecting samples was intolerable as feces are excreted and volunteers who serve as FMT donors must overcome psychological and physical obstacles in order to collect samples.

*S1 (male, 20 years): “Actually, I found the procedure of taking feces and the technique of taking feces difficult to accept at first; after all, it's rather disgusting and it's excreta, so I'd be ashamed if other people found out.”*

*S5 (male, 20 years): “I can't get over the sampling process. When I first went to take the sample, I also found it particularly difficult not to breathe every time, and I am also a germaphobe, so when I took the sample, I found the whole sampling process and the smell unacceptable.”*

#### 3.3.5 Cumbersome transportation procedures

Some donors believe that the lengthy procedure is a significant barrier to fecal donation. Hospitals will perform extensive medical examinations on donors both prior to and during the donation process in order to ensure the quality and safety of donated stool material.

*S10 (female, 20 years): “I feel that the location of the medical checkups is a bit far away because the audience of the volunteers of the FMT supply is the university campuses, and some students are lazy to go to the hospitals because of the far distance of the medical checkups, and there are similar volunteer activities before, but the medical checkups were held nearby the schools, so I think more people may participate in this way.”*

*S21 (female, 23 years): “I found the whole process of donating to be quite complicated with many steps, which greatly diminished my enthusiasm to participate. For example, ice pack storage…”*

#### 3.3.6 Insufficient feedback on medical examinations

Some respondents stated that delayed or insufficient feedback from healthcare personnel was another factor that reduced participants' willingness to continue donating.

*S13 (female, 19 years): “I felt that the officer in charge should have provided the results of the medical examination quickly, as I was not well informed about many of the indicators tested.”*

*S23 (male, 19 years): “I think the quick feedback from the medical reports will help improve future routines.”*

## 4 Discussion

This study investigated the motivations and barriers affecting university students' participation in fecal donation. The findings highlighted several key factors influencing their decisions, which are crucial for developing effective strategies to recruit and retain fecal donors.

Curiosity about FMT, altruism, and the opportunity to contribute to scientific research emerged as closely related intrinsic motivations. Participants' interest in learning about an unfamiliar medical intervention often coexisted with a desire to help patients and support medical advancement. This pattern is consistent with previous studies showing that participation in medical donation and health research is frequently shaped by knowledge-seeking, prosocial values, empathy, and perceived social contribution (27, 28). Recruitment messages should therefore explain both the scientific basis of FMT and the potential contribution of fecal donation to patient care and medical research.

Financial compensation and free health examinations represented important extrinsic motivations. For university students with limited disposable income, compensation may offset the time, inconvenience, and transportation costs associated with repeated screening and donation. Free health assessments may also provide reassurance and personal health information that participants might otherwise obtain at their own expense. Similar motivations have been reported in studies of biospecimen and blood donation (18, 29). Nevertheless, compensation should remain proportionate to participants' time and inconvenience, because excessive financial incentives could create undue inducement or encourage concealment of temporary health problems that might compromise recipient safety.

Social stigma, psychological discomfort, and disruption to daily life formed an interconnected group of barriers (16). Participants described embarrassment, disgust, concerns about privacy, negative reactions from family members or peers, and conflicts with academic or daily schedules. These findings are consistent with multicenter evidence showing that limited knowledge, stigma, inconvenience, and discomfort with stool handling can deter potential donors before enrolment. Stool donation may be particularly sensitive because feces are commonly associated with privacy concerns and social discomfort (17). Recruitment programs should therefore normalize discussion of fecal donation, protect confidentiality, provide clear collection instructions, use supportive communication, and offer flexible scheduling to reduce embarrassment, anxiety, and disruption to daily life.

Stringent eligibility criteria, repeated testing, transportation difficulties, and delayed feedback also reduced willingness to continue. Although rigorous screening is essential for recipient safety, avoidable organizational burdens may contribute to attrition. Previous studies have shown that social history, body mass index restrictions, repeated laboratory examinations, and logistical inconvenience exclude or discourage many potential donors (15, 30). Donor programs should therefore maintain safety standards while improving appointment coordination, transportation support, communication, and the timeliness of medical-report feedback. Importantly, all participants in the present study had successfully completed the donation process. The barriers identified here therefore represent challenges that successful donors were able to tolerate or overcome and may not capture the more decisive reasons for refusal, screening failure, or early withdrawal among non-participants. Future studies should include individuals at each stage of the donor pathway. Practical strategies may include establishing partnerships with universities, providing campus-based education and consultation, improving transportation arrangements, coordinating screening appointments, offering timely medical-report feedback, and maintaining regular communication with donors.

## 5 Strengths and limitations

A strength of this study was the use of in-depth interviews, maximum-variation purposive sampling, and collaborative thematic analysis to explore the experiences of university students with diverse demographic and academic backgrounds. Nonetheless, there are some limitations to the study. First, this study was conducted at a single university campus in eastern China, and the sample consisted exclusively of university students who had successfully completed the entire fecal donation process. The findings may therefore reflect the educational, institutional, cultural, and socioeconomic context of this particular campus and may not be directly transferable to students at other universities, non-academic young adults, or socioeconomically diverse youth populations across China. Second, the inclusion of only successful volunteers introduces selection bias. The identified obstacles mainly represent challenges that participants were able to navigate or tolerate and may not reflect the lived experiences or more decisive barriers of students who declined participation, failed screening, or withdrew early. Consequently, the obstacle-related findings should not be generalized to all potential fecal donors. Future multicenter studies should include non-participants, screening failures, and early dropouts to provide a more comprehensive understanding of barriers across the entire donor pathway. Third, the decision-making needs in the study were identified through semi-structured interviews, which, although reflecting the main motivations of college student volunteers, may not cover all motivations and needs. Future studies may consider using quantitative research methods to obtain more comprehensive data. Finally, two interviews were conducted by telephone, which may have limited the researchers' ability to observe non-verbal cues and capture the contextual richness available in face-to-face interviews.

## 6 Conclusion

This study identified five motivations and six barriers influencing university students' participation in fecal microbiota donation. Motivations included interest in FMT and its potential benefits, altruism, financial compensation, free health examinations, and the opportunity to contribute to scientific research. Barriers included negative perceptions, disruption to daily life, stringent donor eligibility requirements, psychological discomfort associated with stool collection, logistical burdens, and insufficient or delayed feedback on medical examinations.

These findings suggest that donor programs may improve recruitment and retention by strengthening education about FMT, providing clear and timely communication, protecting donor privacy, reducing avoidable logistical burdens, and offering appropriate support throughout screening and donation. Because the participants were drawn from a single university and had all successfully completed the donation process, the findings should be interpreted cautiously and should not be generalized to all potential donors. Future multicenter studies should include individuals who decline participation, fail screening, or withdraw early.

## Data Availability

The original contributions presented in the study are included in the article/supplementary material, further inquiries can be directed to the corresponding authors.
